# Its Objects Attained

**Published:** 1920-07-10

**Authors:** 


### THE WINDING-UP OF A NURSING ASSOCIATION.

### Its Objects Attained.

A meeting of the Association for the Promotion of the Registration of Nurses in Scotland was held on July 2 at 8 Drumsheugh Gardens, Edinburgh, when the affairs of the Association were finally wound up, its objects having been attained by the passing into law of the Nurses' Registration Acts. Sir James Affleck, who presided, said the Association was founded about eleven years ago, and from a small beginning it had grown to have a membership of over 3,000. He had no doubt that it was due in no small measure to the advocacy which was carried on by their Association that the Bill was brought before Parliament, and that the State Registration of Nurses was secured. He referred to the great loss which the Association had sustained through the death of its President, the late Lord Inverclyde.

The Hon. Secretary, Dr. D. J. Mackintosh, C.B., M.V.O., gave a short *résumé* of the objects of the Association. He pointed out that the movement in Scotland to secure Registration of Nurses by the State was inaugurated in 1908, when it was found that the promoters of a Bill for the Registration of Nurses, which applied particularly to England, were not prepared to accord any consideration to Scottish needs. This Association, after very serious consideration and with the greatest reluctance, moved for a separate Registration Bill, although they fully recognised that one Central Register for the three Kingdoms was more desirable. A number of influential medical men, hospital matrons, and trained nurses interested themselves in the movement. A Scottish Bill was drafted, and, like the other Bill, was introduced into the House of Commons and passed its first reading. The subject being one of national importance, a Central Committee was formed to see if a general scheme could be framed that would meet the needs of the United Kingdom and one Bill agreed upon. Many conferences took place, but no agreed Bill was presented to Parliament. Some years elapsed, and two further Registration Bills were drafted, one by the Central Committee for the State Registration of Nurses, and another by the College of Nursing, which was established in 1916. Repeated attempts were made by these two bodies to come to terms in order to have an agreed Bill, but without result, when the Minister of Health, on behalf of the Government, decided to introduce a Bill in the House of Commons. On November 6, 1919, the Government introduced a Bill for the State Registration of Nurses, which received the Royal Assent on December 23, 1919. It was found necessary to have a separate Bill for England and Wales, one for Scotland, and one for Ireland, but, with a view to securing a uniform standard of qualification in all parts of the United Kingdom, the Scottish Nursing Council must consult with any Nursing Councils which may be established by Parliament for England and Wales or Ireland respectively. The object of this Association, as I have stated, was to procure State Registration of Nurses, and largely through its efforts this has now been accomplished.

Professor Glaister moved: "That the Association be now dissolved, and that the credit balance in the hands of the Treasurer, amounting to £1 11s. 4d., be handed to the Treasurer of the King Edward VII. Memorial Home for Nurses in Scotland."

A vote of thanks to the office-bearers, the Hon. Secretary, and the Treasurer was moved by Miss Philp, and Dr. Maxtone Thom proposed a vote of thanks to the Chairman, which were both carried.

